# *In vitro* Trypanocidal Activity, Genomic Analysis of Isolates, and *in vivo* Transcription of Type VI Secretion System of *Serratia marcescens* Belonging to the Microbiota of *Rhodnius prolixus* Digestive Tract

**DOI:** 10.3389/fmicb.2018.03205

**Published:** 2019-01-24

**Authors:** Fabio Faria da Mota, Daniele Pereira Castro, Cecilia Stahl Vieira, Marcia Gumiel, Julia Peixoto de Albuquerque, Nicolas Carels, Patricia Azambuja

**Affiliations:** ^1^Laboratório de Biologia Computacional e Sistemas, Instituto Oswaldo Cruz, Fundação Oswaldo Cruz (IOC/FIOCRUZ), Rio de Janeiro, Brazil; ^2^Instituto Nacional de Ciência e Tecnologia em Entomologia Molecular (INCT-EM), Rio de Janeiro, Brazil; ^3^Laboratório de Bioquímica e Fisiologia de Insetos, Instituto Oswaldo Cruz, Fundação Oswaldo Cruz (IOC/FIOCRUZ), Rio de Janeiro, Brazil; ^4^Laboratório de Enteropatógenos, Microbiologia Veterinária e de Alimentos, Departamento de Microbiologia e Parasitologia, Instituto Biomédico, Universidade Federal Fluminense (MIP/UFF), Rio de Janeiro, Brazil; ^5^Laboratório de Modelagem de Sistemas Biológicos, National Institute for Science and Technology on Innovation in Neglected Diseases (INCT-IDN), Centro de Desenvolvimento Tecnológico em Saúde, Fundação Oswaldo Cruz (CDTS/FIOCRUZ), Rio de Janeiro, Brazil

**Keywords:** *Serratia marcescens*, *Rhodnius prolixus*, *Trypanosoma cruzi*, trypanocidal activity, antagonistic genes

## Abstract

*Serratia marcescens* is a bacterium with the ability to colonize several niches, including some eukaryotic hosts. *S. marcescens* have been recently found in the gut of hematophagous insects that act as parasite vectors, such as *Anopheles, Rhodnius*, and *Triatoma*. While some *S. marcescens* strains have been reported as symbiotic or pathogenic to other insects, the role of *S. marcescens* populations from the gut microbiota of *Rhodnius prolixus*, a vector of Chagas’ disease, remains unknown. Bacterial colonies from *R. prolixus* gut were isolated on BHI agar. After BOX-PCR fingerprinting, the genomic sequences of two isolates RPA1 and RPH1 were compared to others *S. marcescens* from the NCBI database in other to estimate their evolutionary divergence. The *in vitro* trypanolytic activity of these two bacterial isolates against *Trypanosoma cruzi* (DM28c clone and Y strain) was assessed by microscopy. In addition, the gene expression of type VI secretion system (T6SS) was detected *in vivo* by RT-PCR. Comparative genomics of RPA1 and RPH1 revealed, besides plasmid presence and genomic islands, genes related to motility, attachment, and quorum sensing in both genomes while genes for urea hydrolysis and type II secretion system (T2SS) were found only in the RPA1 genome. The *in vitro* trypanolytic activity of both *S. marcescens* strains was stronger in their stationary phases of growth than in their exponential ones, with 65–70 and 85–90% of epimastigotes (Dm28c clone and Y strain, respectively) being lysed after incubation with RPA1 or RPH1 in stationary phase. Although T6SS transcripts were detected in guts up to 40 days after feeding (DAF), *R. prolixus* morbidity or mortality did not appear to be affected. In this report, we made available two trypanolytic *S. marcescens* strains from *R. prolixus* gut to the scientific community together with their genomic sequences. Here, we describe their genomic features with the purpose of bringing new insights into the *S. marcescens* adaptations for colonization of the specific niche of triatomine guts. This study provides the basis for a better understanding of the role of *S. marcescens* in the microbiota of *R. prolixus* gut as a potential antagonist of *T. cruzi* in this complex system.

## Introduction

*Trypanosoma cruzi* is a protozoan parasite that causes Chagas disease (also known as American Trypanosomiasis), a neglected disease that affects six to seven million people worldwide and is transmitted by triatomines, which are insect vectors from the family of Reduviidae (Coura, 2015; World Health Organization [WHO], 2018). *T. cruzi*, after been ingested by a triatomine vector, multiplies as epimastigotes within its midgut and finally in the rectum where it differentiates into infective metacyclic trypomastigotes, which can be eliminated through feces during the triatomine blood meal on the vertebrate host (Cortez et al., 2012; Ferreira et al., 2016). Although *T. cruzi* transmission through triatomine vectors is considered the principal infection mechanism, the oral route transmission through food contaminated by infected triatomines is increasing, especially in the Amazon region, including Brazil, Colombia, Venezuela, French Guyana, and Bolivia (Noya and González, 2015).

Chagas disease presents an acute phase with mild or no symptoms and a chronic phase during which parasites can be hidden mainly in the heart and digestive muscles (Coura and Viñas, 2010). As the infection proceeds, it leads to sudden heart failure due to the progressive destruction of nervous connections. Chagas disease has been reported mainly in Latin America where it is endemic in 21 countries, but over the past decades, it has also been detected in the United States, Canada, Europe, and some Western Pacific countries (Coura and Viñas, 2010; World Health Organization [WHO], 2018) due to infected people emigration and blood transfusion (Hernández-Romano et al., 2015). Because of the lack of a vaccine, vector control plays a key role in the prevention of Chagas disease, which is performed through the use of insecticides. However, insecticide resistance has been extensively detected over the last 15 years (Vassena et al., 2000) and deserves attention because of its increasing impacts on costs of vector control (Germano et al., 2012) and of its interference with the dynamic of triatomine population *in situ*. As a result, the re-infestation by triatomines of different species, such as *Triatoma brasiliensis* and *Triatoma pseudomaculata*, infected with *T. cruzi*, has been notified in endemic areas to replace *Triatoma infestans*, the original triatomine vector (Coura and Viñas, 2010; Pessoa et al., 2015; Barbosa-Silva et al., 2016).

A sustainable alternative strategy to insecticide is biological control. Fungi (Vázquez-Martínez et al., 2014; Forlani et al., 2015; Garcia et al., 2016), and toxin-producing bacteria such as *Bacillus thuringiensis* are already largely used as commercial biopesticides. The biological control by symbiotic bacteria, such as *Wolbachia* sp., has also been demonstrated to be effective in the control of *Aedes aegypti* (McMeniman et al., 2009; Bian et al., 2010; Hancock et al., 2016).

Since 2002, Kondo et al. (2002) demonstrated that the biological control of host’s reproduction with *Wolbachia* is a successful alternative strategy to transgenic approaches. In mosquitos, *Wolbachia* can be transferred between insect species and rapidly spread through natural populations of *A. aegypti* (McMeniman et al., 2009). However, exclusion of *Wolbachia* from the reproductive organs of mosquito vectors by other bacteria such as those from the genus *Asaia* may prevent its control effectiveness (Rossi et al., 2015). In addition, antibiotics produced by the native microbiota of triatomine digestive tract (TDT) also could disturb *Wolbachia* colonization in new vector species (Rossi et al., 2015). Consequently, it is important to know the members of TDT microbiota and their respective functionalities in that environment.

*Rhodococcus rhodnii*, a bacterial symbiont of *Rhodnius prolixus* reported in some specimens bred in insectaries, was suggested to provide the vector with vitamins (Pachebat et al., 2013). Paratransgenic approaches proposed that *R. rhodnii* could be genetically modified and reintroduced in the TDT microbiota to control Chagas disease vectors (Hurwitz et al., 2011) through (i) systemic RNAi (Taracena et al., 2015; Whitten et al., 2016), (ii) the expression of antimicrobial molecules, such as cecropin A (Durvasula et al., 1997; Hurwitz et al., 2012), or (iii) functional antibody fragment (Durvasula et al., 1999).

Gumiel et al. (2015) observed that the TDT microbiota might differ between triatomines from insectary and from the wild. Although actinobacteria such as *R. rhodnii* were reported in some traditional *Rhodnius* insectary colonies (Eichler and Schaub, 2002; Dias et al., 2015), these have been only sporadically observed in specimens from field capture. On the other hand, *Serratia* species has been predominantly diagnosed in the TDT microbiota of wild *Triatoma* specimens, by high-throughput sequencing of ribosomal gene (Gumiel et al., 2015), and in *Rhodnius* from other insectaries by Sanger sequencing (da Mota et al., 2012) as well as by 454 pyrosequencing of 16S rRNA (Vieira et al., 2015). Globally, it seems that bacteria with GC-rich genomes are the dominant microbial components in the ecological niche of TDT (Carels et al., 2017).

*Serratia* sp. is ubiquitous and found widespread around the globe. Members of *Serratia* genus exist in a wide range of habitats, including water, soil, and the digestive tracts of various animals, showing extremely diverse ecological adaptations (Petersen and Tisa, 2013). *Serratia* includes insect symbionts (Manzano-Marín et al., 2016), insect pathogens (Grimont et al., 1979), nematode pathogens (Schulenburg and Ewbank, 2004), plant pathogens (Zhang et al., 2003), plant growth-promoting rhizobacterium (Berg, 2000), coral pathogens (Patterson et al., 2002), nosocomial human pathogens (Mahlen, 2011), and bacterial antagonists (Thomson et al., 2000; Masschelein et al., 2013).

While *R. rhodnii* has been cited as a symbiotic member of TDT, the role of *Serratia* in the TDT environment and in vector homeostasis is not clear. However, there are some pieces of evidence that *S. marcencens* interferes with the success rate of digestive tract colonization by *T. cruzi* in *R. prolixus* (Azambuja et al., 2004; Castro et al., 2012; Vieira et al., 2016). In addition, an entomopathogenic *S. liquefaciens* Strain FK01 (Taira et al., 2014), which was originally isolated from the ant lion, was shown to be highly virulent to the American cockroach and the silkworm (Egami et al., 2009). Ultrastructures showing the lysis of the human parasite *Leishmania* (*Leishmania*) *chagasi* by *S. marcescens* were also reported by electron microscopy (Moraes et al., 2008).

Different secretions systems were reported in *S. marcescens* isolated from other environments (Alcoforado-Diniz and Coulthurst, 2015). These secretions systems could export toxins or other molecules with high antagonistic potential against other bacterial members of TDT microbiota community or against eukaryotic cells, such as fungi, protozoa, or epithelial cells from the vector itself (Li et al., 2015). Among these bacterial secretion systems, the type VI secretion system (T6SS) fires toxic proteins into target cells like a nanomachine. The target structure can vary depending on the different subassemblies of the T6SS machinery (Gerc et al., 2015). The T6SS represents a means by which bacteria interact with their host or attack competitors organisms by contact with an attacking neighbor cell. Azambuja et al. (2004) suggested that prodigiosin could be an important factor contributing to the trypanolytic action of *Serratia*. Castro et al. (2007a) also suggested that bacterial fimbriae could participate to the lysis of protozoan parasites induced by *S. marcescens*, once the trypanolytic effect of *S. marcescens in vitro* was dependent on D-mannose and distinct from the hemolytic activity. However, the genomic diversity of *Serratia* populations in TDT, as well as the ability of *S. marcescens* strains to express their antagonistic mechanisms *in vivo*, remain unknown. Therefore, the aim of this study was (i) to obtain *S. marcescens* isolates from *R. prolixus* TDT microbiota, (ii) to evaluate their *in vitro* trypanolytic activity, (iii) to acquire their draft genomic sequences, (iv) to suggest genetic determinants in their genomes with antagonist potential against *T. cruzi*, and (v) to detect T6SS transcripts in *R. prolixus* TDT.

## Materials and Methods

### Maintenance of *R. prolixus* Specimens and Ethics Statement

Specimens of *R. prolixus* were maintained, under controlled temperature and humidity, in a colony at *Laboratório de Bioquímica e Fisiologia de Insetos, Instituto Oswaldo Cruz*. The triatomines were fed through an artificial apparatus with defibrinated rabbit blood provided by the *Instituto de Ciência e Tecnologia em Biomodelos* (ICTB) (Azambuja and Garcia, 1997). The rabbit blood was obtained according to the ethical principles in animal experimentation approved by the *Comissão de Ética no Uso de Animais* from Oswaldo Cruz Foundation (CEUA/FIOCRUZ) under the protocol number LW019/17 following the recommendations of *Ministério da Ciência, Tecnologia e Inovação*/*Conselho Nacional de Controle de Experimenyação Animal* (MCTI/CONCEA) available at http://pages.cnpem.br/ceua/wp-content/uploads/sites/56/2015/06/DBCA.pdf, which is approved by the *Federation of European Laboratory Animal Science Associations* (FELASA), the *American Association for Animal Science* (AAAS), the *Association for Assessment and Accreditation of Laboratory Animal Care International* (AAALAC), and the *International Council for Animal Science* (ICLAS).

### Multiplication of *T. cruzi in vitro* and *in vivo*

The *T. cruzi* Dm28c clone (Contreras et al., 1988) and *T. cruzi* Y strain (Silva and Nussenzweig, 1953), previously classified as TcI and TcII, respectively (Zingales et al., 2009), were supplied by Dr. Otacílio Moreira (*Laboratório de Biologia Molecular de Doenças Endêmicas*, Fiocruz, Brazil). Epimastigotes of *T. cruzi* were grown *in vitro* in brain heart infusion (BHI) media (Sigma-Aldrich) containing folic acid (30 mg/L), hemin (25 mg/L), and supplemented with 10% heat-inactivated fetal bovine serum (FBS) at 28°C (Azambuja and Garcia, 1997). The epimastigotes parasites in the exponential growth phase were used for further *in vivo* and *in vitro* assays. The number of parasites was quantified using an optical microscope in a Neubauer chamber and diluted to concentrations of 2.5 × 10^6^ epimastigotes/mL for *in vitro* assay and 1 × 10^7^ epimastigotes/mL for triatomine infection (*in vivo* assay). The Mann–Whitney statistical test was used to verify whether there were any statistically significant differences in the parasite number from TDTs.

### Infection of *R. prolixus* Specimens by Oral Administration of *T. cruzi*

Fifth instar nymphs were randomly picked up and fed with defibrinated rabbit blood containing *T. cruzi* epimastigotes of the Y strain or Dm28c clone. The blood complement system was previously heat-inactivated by centrifugation at 1890 × *g* for 15 min at 4°C and plasma (supernatant) incubated for 30 min at 55°C. Subsequently, erythrocytes previously washed with phosphate buffered saline (PBS; 0.15 NaCl in 0.01 M sodium phosphate buffer, pH 7.2) were mixed with plasma treated as described above and *T. cruzi* were added at a final concentration of 1 × 10^7^ epimastigotes/mL to the reconstituted blood. Uninfected individuals (control) were fed only on inactivated blood without parasites. Only fully engorged fifth instar *R. prolixus* nymphs were used for the experiments.

### Dissection of *R. prolixus* Digestive Tract

At 1, 2, 7, 15, 30, and 40 days after feeding (DAF), fifth instar nymphs of *R. prolixus* were washed to eliminate surface bacteria with 70% ethanol and several times with sterile distilled water. All dissections were performed under aseptic conditions. For parasite quantification in insect digestive tract, the abdomen cuticle was removed and the contents of the whole digestive tract were collected in sterile Eppendorf tubes, containing PBS at a proportion of five stomachs or intestines/500 μL PBS. To perform RNA extraction, three pools containing each one five (stomach) or posterior (intestine) midgut samples were collected and stored in sterile 1.5 mL microtubes at -70°C (Vieira et al., 2016).

### Bacterial Colony Isolation

In order to obtain single colonies, the content of stomach lumens was serially diluted, spread onto the surface of BHI agar, and incubated for 48 h at 28°C. Thirty colonies were randomly picked up and subcultured at 28°C for 48 h on BHI agar.

### Extraction of Genomic DNA From Bacterial Isolates

Genomic DNAs were isolated using a Wizard genomic DNA purification kit, according to the manufacturer’s instructions. For each bacterial isolate, the cells from 10 mL cultures grown in BHI medium at 28°C for 24 h were centrifuged at 10,000 × *g* for 10 min.

### Molecular Typing of Bacterial Isolates by BOX-PCR Genomic Fingerprinting

Amplification reactions with BOXA1R primer were performed as described by da Mota et al. (2002) in a mix containing 600 μM of each dNTP, 3.75 mM MgCl_2_, 50 mM KCl, 20 mM Tris–HCl (pH 8.4), 1 μM of the primer BOXA1R (5′-CTACGGCAAGGCGACGCTGACG-3′), about 50 ng of genomic DNA and 1.25 U of *Taq* DNA polymerase. The PCR conditions were comprised of an initial denaturation step for 7 min at 95°C followed by 30 cycles of denaturation at 94°C for 1 min, annealing at 53°C for 1 min, and extension at 65°C for 8 min, with a final elongation step of 16 min at 65°C. The PCR products were analyzed by electrophoresis in 1.4% agarose gel in Tris–borate–EDTA buffer for 3 h 30 min, at 90 V and room temperature.

### Trypanolytic Activity of Bacterial Isolates *in vitro*

Bacterial isolates with different fingerprinting profiles were previously cultured in liquid BHI medium for 18 h at 30°C. Then, 10 microliters of each culture were aseptically sub cultured into 5 mL of sterile BHI and incubated at 30°C for 2 h (exponential phase) or 20 h (stationary phase) and 90 rpm. Since the antagonistic effect of *S. marcescens* over *T. cruzi* occurs through trypanolysis, aliquots collected from these bacterial cultures were used in a trypanolytic assay *in vitro* with *T. cruzi* Dm28c and Y epimastigotes, as previously described by Azambuja et al. (2004) and Castro et al. (2007b). Bacterial suspensions in the concentration of 1 × 10^8^ CFU/mL were added to 80 μL of *T. cruzi* Dm28c clone and Y strains epimastigote suspensions at the final concentration of 2.5 × 10^6^ parasites/mL in Eppendorf tubes, and incubated at 30°C for 120 min. The whole procedure was carried out under aseptic conditions and incubated for 2 h at 30°C. In each assay, the living parasites, predictable by their apparently un-affected morphology and at least some slight flagellar movements, were counted using a light microscope and a Neubauer hemocytometer chamber. The control of each assay was obtained by adding 10 μL of PBS in the medium instead of a bacterial suspension. The experiment was repeated twice with around ten incubations for each group. The trypanolytic activity was expressed as the ratio (%) of lysed parasites to the number of parasites in the control after 2 h of incubation. The one-way ANOVA statistical test was used to determine whether there were any statistically significant differences between the samples incubated with bacteria or without bacteria (control). Moreover, we tested the statistical significance of differences between averages through *t* test.

### Genomic Sequencing of Bacterial Isolates

The trypanolytic strains, RPA1 and RPH1 had two different BOX fingerprinting profiles and were deposited in the *Coleção de Enterobacterias* – CENT at the *Fundação Oswaldo Cruz* (Fiocruz), Brazil. Whole-genome sequencing (WGS) of these strains were performed using an Illumina HiSeq 2500 sequencer from the high-throughput sequencing platform of Fiocruz. Reads were *de novo* assembled using the SPADES (version 3.1.1) assembler. Gene prediction and annotation were performed with the Prokaryotic Genome Automatic Annotation Pipeline (version 1.11). The Whole Genome Shotgun projects have been deposited at DDBJ/ENA/GenBank under the accession NCQJ00000000 and NCQI00000000. The versions described in this paper are versions NCQJ01000000 and NCQI01000000, respectively, for RPA1 and RPH1 strains.

### Genomic Average Nucleotide Identity Between *S. marcescens* Strains

The average nucleotide identity (ANIm) was calculated using the NUCleotide MUMmer software (version 3.1) for RPA1, RPH1, and others 377 genomic sequences of *Serratia marcescens* available at RefSeq NCBI https://www.ncbi.nlm.nih.gov/genome/genomes/1112, accessed on 05/29/2018.

### Mapping and Comparative Genomics of *S. marcescens* Strains

The BLAST Ring Image Generator (BRIG) software was used to generate genomic maps that show multiple prokaryote genome comparisons among the RPA1, RPH1, and their closest genomes based on their ANIm. The closest genomic sequences for the RPA1 strain were sicaria-Ss1, ADJS-2D_White, SOLR4, and RSC-14, while for the RPH1 strain they were 19F, S2I7, WW4, and EGD-HP20, as shown in the spreadsheet called “ANIm *Serratia* strains” from the Supplementary Material.

### Prediction of Antimicrobial Resistance Genes and Genomic Islands

Antimicrobial resistance genes were searched in draft genomic sequences of *S. marcescens* RPA1 and RPH1 using KmerResistance (Clausen et al., 2016) while the putative genomic islands related to antibiotic resistance, metabolism, pathogenicity, or symbiotic lifestyle were found using GIPSy (Soares et al., 2016). Genomic islands and operons were visualized on genome sequences with BRIG and ARTEMIS, respectively.

### Bacterial Secretion Systems and Primer Design for T6SS Genes of *S. marcescens*

All protein sequences predicted from coding sequences (CDS) from draft genomes of *S. marcescens* RPA1 and RPH1 strains were compared to *nr* database (non-redundant protein NCBI database, release 72) and analyzed by MEGAN (version 5.11.3) in order to identify the proteins related to bacterial secretion systems.

The sequences of ClpV (a gene involved in T6SS recycling, Douzi et al., 2016), Hcp1 (a key hexameric protein of the tubular T6SS secretion system, Brunet et al., 2014), and DotU1 (encoding homologs of T4SS stabilizing proteins, Bröms et al., 2012) that encode key proteins of T6SS were used to design primers, as shown in the spreadsheet called “SecretionSystems” from the Supplementary Material.

### *In vivo* Detection of T6SS Gene Expression and the Constitutive Bacterial *rpoB* Gene by RT-PCR

Triatomines at 1, 7, 15, and 40 DAF (*T. cruzi* infected and non-infected) were dissected to prepare pools of five stomachs and intestines, as previously described (Vieira et al., 2016). Total RNA was obtained using the NucleoSpin^®^RNA II Kit (Macherey-Nagel, Düren, Germany) following the manufacturer’s instructions and quantified using a NanoDrop 2000 Spectrophotometer (Thermo Scientific, Waltham, MA, United States). Synthesis of cDNA was performed with a First-Strand cDNA Synthesis Kit (GE Healthcare, Buckinghamshire, United Kingdom) following the manufacturer’s protocol using 2.5 μg of total RNA and the pd(N)6 primer. cDNA was quantified by fluorescence using a Qubit Fluorimeter (Life Technologies) with the ssDNA assay kit. Here, we analyzed the gene expression of three genes for T6SS of *S. marcescens* (ClpV, Hcp, and DotU) and the constitutive *rpoB* gene, which encodes the β subunit of bacterial RNA polymerase. The following primers were designed based on the sequences of RPA1 and RPH1 genomes for the amplification of the gene fragments: ClpV_F1692-1718(5′-GGT GkT GkC sGA yTG GAC CGG CAT CCC-3′) and ClpV_R2158-2183(5′-CCG TCy TCC ATC mmG CCC TTG TCG AA-3′); DotU_F264-290(5′-CGG GCT GGT GAC CTT CCA TAA CGA-3′) and DotU_R792-815(5′-CAC GCT TTG CTG CGC TTC GTC GCG-3′); Hcp_F26-51(5′-TGT TCC TGA AAG TTG AAG GTG CCA GC-3′); Hcp_R489-515(5′-CGG TTT TCC TTG ATG TTC CAA CCT GCG-3′). Previously designed primers by Dahllöf et al. (2000) were used for *rpoB* constitutive gene, rpoB_1698f (5′-AAC ATC GGT TTG ATC AAC-3′) and rpoB_2041r (5′-CGT TGC ATG TTG GTA CCC AT-3′). Each singleplex reaction was run for each pool of triatomines (*n* = 3) and contained 10 ng cDNA, primer pairs (0.2 μM), MgCl_2_ (2.5 mM), dNTPs (200 μM each), buffer and GoTaq^®^DNA polymerase (0.6 U) from Promega at a final volume of 25 μl. The cDNA was amplified at 95°C for 5 min followed by 38 cycles of 95°C for 40 s, 52°C for 1 min and 72°C for 40 s, with a final extension for 7 min at 72°C. Negative controls of PCR reactions were carried out without cDNA template in order to assess dimer formation between primers or reaction contaminations by foreign DNA. The agarose gel electrophoresis of PCR products was performed with 1.4% agarose in Tris–borate–EDTA buffer at 100 V for 2 h at room temperature.

## Results

### Isolation of Bacterial Colonies From *R. prolixus* Gut and BOX-PCR Genomic Fingerprinting

**FIGURE 1:**
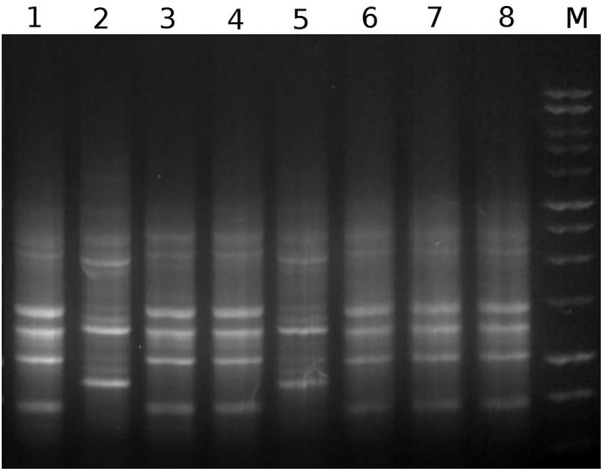
A representative example of genomic fingerprints generated by separation of BOXA1R amplicons through agarose gel electrophoresis. The numbers 1–8 represent BOXA1R profiles obtained with the isolates RPA1, RPH1, RPD1, RPE1, RPG11, RPC11, RPD4, and RPB1, respectively. M represents the 1 kb DNA ladder from Promega.

We observed distinct types of morphologies as well as colors (white and red) among the colonies isolated from the *R. prolixus* gut microbiota and sub cultured on BHI agar plate (data not shown). Molecular fingerprinting of their genomic DNA revealed that these isolates belong to at least two distinct genomic profiles (Figure 1). BOXA1R profiles similar to those of RPA1 and RPH1, corresponded to about 73 and 26% of isolates, respectively. RPA1 and RPH1 isolates also showed differences in the morphologies of their bacterial colonies on BHI agar. While RPA1 presented white colonies, RPH1 showed strong red colonies, suggesting the synthesis of prodigiosin by this strain.

### Trypanolytic Activity and Antagonism of *S. marcescens in vitro*

**FIGURE 2:**
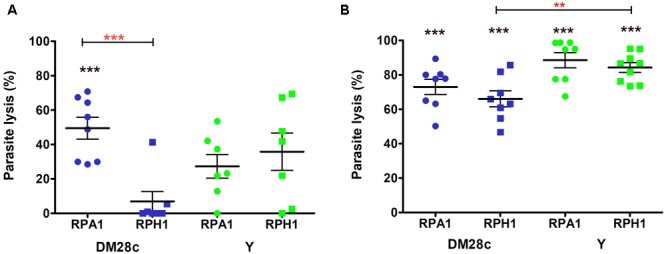
Trypanolytic activity during the exponential **(A)** and stationary **(B)** phases of *S. marcescens* cultures (RPA1 and RPH1 are represented by circles and squares, respectively) with respect to epimastigotes of *T. cruzi* Dm28c (blue) and Y strains (green) after incubation for 2 h at 30°C. The vertical axis represents the percentage of lysed parasites/mL in comparison to the controls without the bacteria (0%). The red asterisks above the bars indicate statistical significances at *p* = 0.0035 (^∗∗^) and *F* = 2.377 or *p* = 0.0003 (^∗∗∗^) and *F* = 1.358 differences between averages using *t* test. The black asterisks (^∗∗∗^) indicate statistical significances at *p* < 0.001 for average differences between the control without bacteria and the group in one-way ANOVA (see details in Supplementary Material).

The trypanolytic activity of both RPA1 and RPH1 *S. marcescens* strains was stronger in their stationary phases of growth than in their exponential ones. In exponential phase (Figure 2A, blue), the percentage of *T. cruzi* Dm28c lysis was significantly higher under incubation with RPA1 (50%) than with RPH1 (7%), on average (Figure 2A; *p* = 0.0003; *F* = 1.358). In their exponential phase, RPA1 and RPH1 isolates showed similar lysis percentages for *T. cruzi* Y, which was about 30–40% (Figure 2A, green). In stationary phase, the percentage of epimastigotes of *T. cruzi* Y that were lysed by both RPH1 and RPA1 was significantly higher with an average of 84 and 88%, respectively (Figure 2B, green). The percentage of epimastigotes of *T. cruzi* Dm28c that were lysed was also significantly higher, on average, when incubated with RPH1 (67%) and RPA1 (73%) in stationary phase (Figure 2B, blue). *T. cruzi* Y was significantly more sensitive to RPH1 in the stationary phase than *T. cruzi* Dm28c (Figure 2B; *p* = 0.0035; *F* = 2.377).

### Genomic Sequences and Genomic Average Nucleotide Identity (ANIm)

The WGS reads were *de novo* assembled using the SPADES (version 3.1.1) assembler, which yielded a dataset composed of 60 contigs, totaling ∼5.3 Mb, with an average G+C content of 59.32% for strain RPA1 and 35 contigs, totaling ∼5.1 Mb, with an average G+C content of 59.63% for strain RPH1.

**FIGURE 3:**
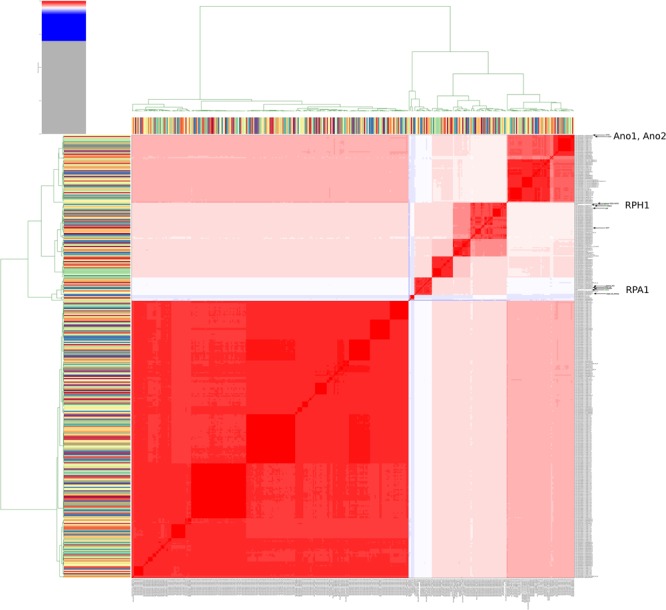
Heatmap of ANIm percentage identity for 379 genomes of *S. marcescens* obtained from RefSeq. White and red cells in the heatmap corresponded to ANIm sequence identity higher than 95% and therefore to the same species (Richter and Rosselló-Móra, 2009), while blue cells correspond to ANIm lower than 95%. The hierarchical clustering follows a two dimension dendrograms organized according to simple linkage of ANIm percentage identities. The analysis indicates up to six strains clades along the heatmap diagonal. The RPA1 and RPH1 strains (isolated from *R. prolixus* gut) are found in different clades, suggesting distinct evolutionary origins and refuting the hypothesis of clonality for these strains. The arrows show the strains that are the closest to RPA1 (sicaria-Ss1, ADJS-2D_White, SOLR4, and RSC-14), RPH1 (19F, S2I7, WW4, and EGD-HP20), and ano1 according to ANIm percentage identity. Ano1 and Ano2 were previously isolated from the gut lumen of adult females of *A. stephensi* mosquitoes (see details and high resolution image in Supplementary Material).

In order to globally evaluate the genomic status of RPA1 and RPH1 genomes according to all genome sequences of *S. marcescens* available in the NCBI repository, we calculated the ANIm for 379 genomes (Supplementary Material, ANI *Serratia* strains). The ANIm analysis indicated up to six clades of *S. marcescens* (red squares, ANIm ≥95%) along the heatmap diagonal (Figure 3). The RPA1 and RPH1 strains (isolated from *R. prolixus* gut) are found in distinct red clades, suggesting that both are *S. marcescens*, but from distinct evolutionary origins and refuting the clonal hypothesis for these strains. The RPA1 genome was closer to sicaria-Ss1 (*Apis mellifera*), ADJS-2D_White (*Scapteriscus borellii*), SOLR4 (*Solanacea*), and RSC-14 (*Solanum nigrum*), while RPH1 strain was closer to 19F (*Atelopus zeteki*), S2I7 (oil contaminated soil), WW4 (paper machine), and EGD-HP20 (wastwater), according to their ANIm percentage identity. Two strains (ano1 and ano2) previously isolated from the gut lumen of adult female *Anopheles stephensi* mosquitoes were found in another distinct clades as shown by arrows in Figure 3.

### Mapping and Comparative Genomics Between *S. marcescens* Strains

**FIGURE 4:**
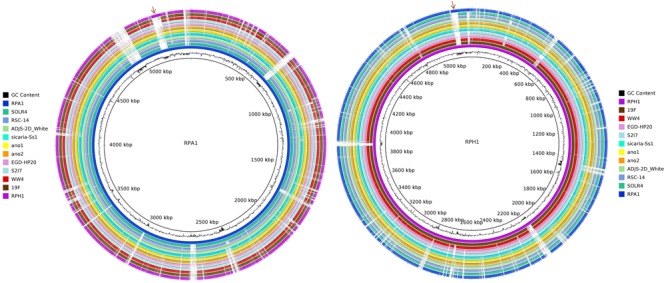
Circular representation of two *S. marcescens* genomes. The CG content bias was plotted as the second internal circle for RPA1 and RPH1 genomes. The brown arrows represent a plasmid shared by RPA1 and RPH1 that includes genes also found in other animal-associated strains. The stronger colors represent the higher blastn identity for the draft genome sequences of RPA1 and RPH1 strains with their closest complete genomes in RefSeq-NCBI based on ANIm, i.e., sicaria-Ss1 (*A. mellifera*), ADJS-2D_White (*S. borellii*), SOLR4 (*Solanacea*), RSC-14 (*Solanum nigrum*) for RPA1, 19F (skin of *A. zeteki*), S2I7 (soil), WW4 (paper mill process waters), and EGD-HP20 (wastwater) for RPH1, as well as the ano1 and ano2 strains previously isolated from the gut lumen of adult female of *A. stephensi* mosquitoes.

Genomic maps of RPA1 and RPH1 strains showed that although they have a high identity with other RefSeq strains, each strain has unique gene islands (Figure 4). Genomic mapping also revealed that strains associated to plants and animals were closer to RPA1 than those associated to free living ones. Some gene islands shared by strains RPA1 and RPH1, suggests that these strains with different evolutionary origins could have recently acquired these gene islands, possibly after niche adaptation. RPA1 has a plasmid highly similar to that of RPH1 (indicated by the brown arrows of Figure 4), which has genes also found in animal-associated strains (ADJS-2D_White, sicaria-Ss1, 19F, ano1, and ano2) but not in plant-associated strains (SOLR4 and RSC-14).

### Gene Prediction, Genomic Islands, and Genome Annotation

The identification of CDSs revealed the presence of at least 4,969 putative CDSs, regions of DNA that can potentially be translated into proteins in RPA1 (locus tag prefix B7L32_) while 4,816 putative CDSs were found in RPH1 genome (locus tag prefix B7L62_). Plasmidial CDSs were found in both genomes, RPA1 (B7L32_24230 to B7L32_24595, B7L32_25505 to B7L32_25515 and B7L32_25585) and RPH1 (B7L62_23970 to B7L62_24190, B7L62_24560 to B7L62_24710 and B7L62_24795). Plasmid stability is probably maintained due to some RelE toxin genes found in the chromosome of each strain (B7L32_21785, B7L32_16920, and B7L32_11945 for RPA1; B7L62_19950, B7L62_13775, and B7L62_06530 for RPH1), while the gene for RelB antitoxin (B7L32_24540 and B7L62_24620) was found only in their plasmids. In addition, a copy of RelE toxin gene (B7L32_25460) was also found in the RPA1 plasmid. Moreover, genes for other complete system toxin–antitoxin (PemK toxins: B7L32_24575 and B7L62_24585 as well as their PemL antitoxins: B7L32_24580 and B7L62_24580) were observed in their plasmids. Among plasmidial CDSs, we also found conjugation genes, including *tra* genes.

**FIGURE 5:**
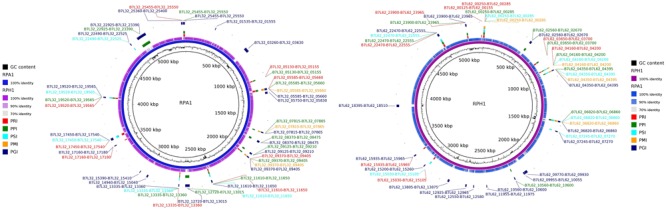
Genomic Islands on RPA1 and RPH1 *S. marcescens* draft genomes and their corresponding genes. The putative phenotypes were predicted by GIPSy software, [Putative Resistance Island (PRI, red), Putative Pathogenic Islands (PPI, green), Putative Symbiotic Islands (PSI, aqua), Putative Metabolic Island (PMI, orange), Putative Genomic Island (PGI, navy)]. The CG content bias was plotted in internal circle of each genome (black).

Most of GIs found in the RPA1 and RPH1 genomes corresponded to regions with a GC bias (Figure 5), suggesting that they could have been acquired on a recent genomic timescale. These GIs included genes related to biologic functions such as antibiotic resistance, metabolism, pathogenicity, and symbiosis.

Among the gene islands of the RPA1 genome were included genes encoding for (i) filamentous hemagglutinin, (ii) hemolysin transporter protein (ShlB) precursor, (iii) phospholipase D, (iv) teichoic acids translocation permease export system (TagGH), (v) teichuronic acid biosynthesis glycosyltransferase (TuaH), (vi) modular non-ribosomal peptide synthase related to linear gramicidin synthase, (vii) chitinases, (viii) pullulanase secretion protein (PulS), (ix) type 4 prepilin-like proteins leader, (x) type II secretion system MLKJIHGFED, (xi) invasion protein regulator, (xii) firmbria A, (xiii) pilus, (xiv) exotoxin, (xv) endolysin, and (xvi) non-ribosomal peptide synthase, among others (see RPA1genesInGeneIslands and RPH1genesInGeneIslands spreadsheets of the Supplementary Material).

### Identification of Putative Resistance Genes *in silico*

*In silico* KmerResistance and Resfinder methods for resistance genes prediction revealed the presence of different antibiotic classes: aminoglycosides [aadA1, aac(6′)-Ib, and aac(6′)-Ic], tetracyclines [tet(41)], and beta-lactams (blaOXA-9, blaTEM-1A, and blaSST-1) in the RPA1 genome sequence. On the other hand, blaOXA-9 and aadA1 genes were not found in the RPH1 genome (Supplementary Material). However, the genes *aadA1, aac(6*′*)-Ib, blaTEM-1A*, and *blaOXA-9* were found in plasmids (see the ResistanceGenes spreadsheet of the Supplementary Material).

### Identification of RPA1 and RPH1 Genes Orthologous to KEGG Database

**FIGURE 6:**
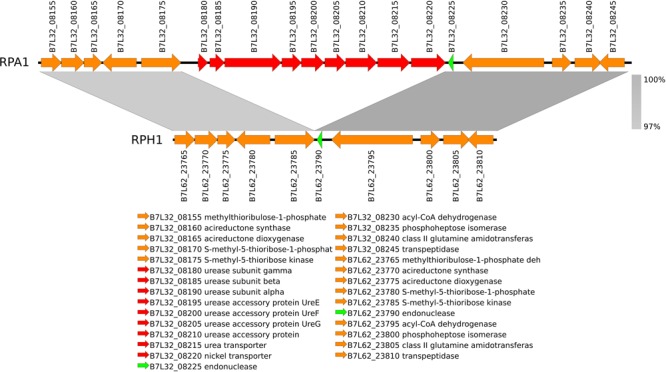
The nickel-dependent urease (EC 3.5.1.5) and accessory genes (red) are included in a gene island found in RPA1 genome, but not in RPH1 strain. This gene island is located between two conserved syntenic flanking regions (gray) characterized by nucleotide similarities higher than 97%.

Ninety genes from the RPA1 genome were absent in RPH1, when annotated by reference to KEGG. We found that they included genes (eight) belonging to urease operon (Figure 6) and fimbriae (see the SharedOrExclusiveKOrthologous spreadsheet of the Supplementary Material). These recent gene acquisitions should be further studied once these genes could bring advantages to *Serratia* considering the specific features of the adaptation niche of hematophagous insects such as *R. prolixus*. On the other hand, RPH1 exhibited 104 genes, including some belonging to multidrug efflux systems, absent in RPA1.

**FIGURE 7:**
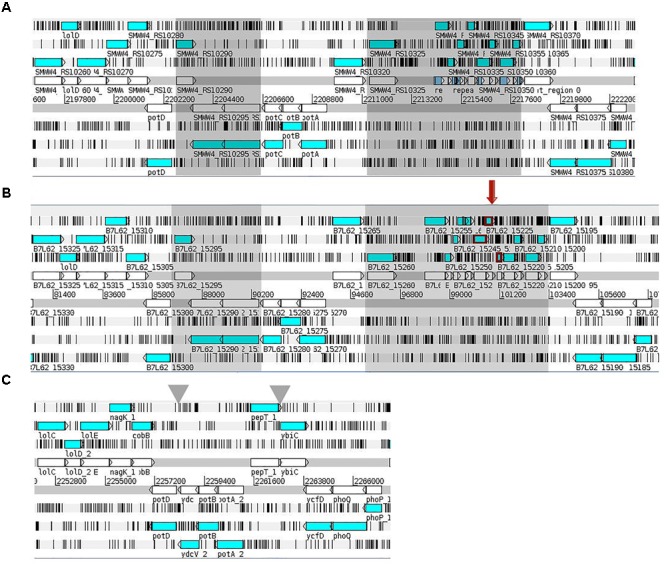
The two genomic regions of *S. marcescens* highlighted by gray rectangles in the WW4 **(A)** and RPH1 **(B)** strains are absent in the RPA1 strain **(C)**. They are localized inside the pot operon (spermidine/putrescine transport system) and between the pepT and ybiC genes. These regions include genes coding for the PTS system maltose-specific EIICB component and the colicin/pyocin bacteriocin operon. The colicin/pyocin bacteriocin operon in RPH1 **(B)** includes a whole pyocin (B7L62_15230), which is partial in WW4; the colicin immunity protein (B7L62_15225) present in RPH1 is absent in WW4 (red arrow). The genes of the two-component regulatory system known as PhoP/PhoQ were present in all strains **(A–C)**.

Another difference between RPA1 and RPH1 is the colicin V/pyocin bacteriocin operon, which is present only in RPH1. In RPH1, the colicin/pyocin operon includes *cda1*, which is a gene encoding colicin-D absent from the WW4 strain (red arrow in Figure 7). Colicin V (ColV) is an antibiotic peptide secreted by some members of the Enterobacteriaceae that enable them to kill closely related bacteria, thereby reducing competition for essential nutrients.

Among the genes shared by RPA1 and RPH1 are those of chitinases, esterases, phospholipid/cholesterol/gamma-HCH transport system, exonucleases, heme acquisition and exporter proteins, iron uptake, transport and chelatases, bicyclomycin/chloramphenicol resistance, microcin C resistance, catalase, peroxidase, chloroperoxidase, nitronate monoxygenase, superoxide dismutases, enterobactin synthetase, pyochelin synthetase, filamentous hemagglutinin, flagellar, secretion system proteins and serralysin, bacillolysin, aquaporin Z, hemolysin, hemolysin III, and virulence factors (see the SharedOrExclusiveKOrthologous spreadsheet of the Supplementary Material).

### Bacterial Secretion Systems

**FIGURE 8:**
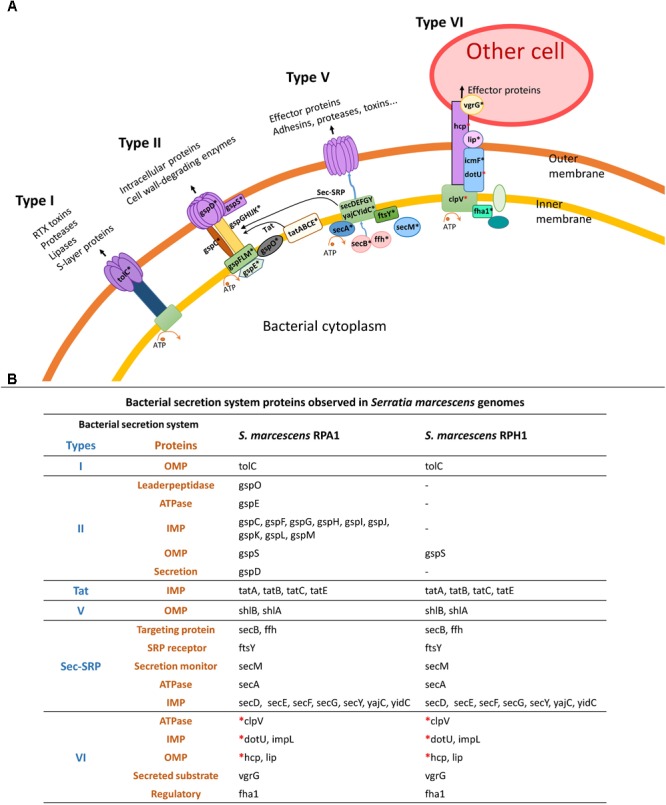
Bacterial secretion systems and their proteins observed in *S. marcescens* RPA1 and RPH genomes isolated from *R. prolixus* gut. **(A)** Schematic illustration of bacterial secretion systems type I, type II, type Vb, and type VI. **(B)** Proteins found in the bacterial secretion systems of *S. marcescens* RPA1 and RPH1 genomes. The red asterisks indicate the genes used for primer design for gene expression analysis *in vivo*. OMP, outer membrane protein; IMP, inner membrane protein; Tat, twin-arginine targeting.

Some bacterial secretion systems were observed in both RPA1 and RPH1 genomes, including Sec-SRP, twin-arginine targeting (Tat), T6SS, and some other genes, such as TolC (an outer membrane protein required for several efflux systems), ShlA (a hemolysin precursor), and ShlB (a hemolysin transporter protein) (Figure 8), while type II system (T2SS) was observed only in RPA1. T6SS is widely used throughout Gram-negative bacteria to inject effector proteins and some toxins in a one-step mechanism directly into the cytosol of the eukaryotic cells being targeted since Hcp proteins can cross the plasmatic membrane of eukaryotic cells (Figure 8) and possibly that of *T. cruzi*.

### T6SS Transcription and Parasite Counting in TDT

**FIGURE 9:**
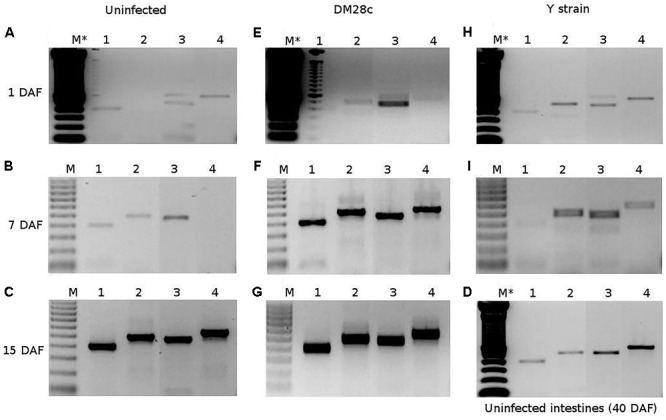
RT-PCR assessment of *in vivo* gene expression of *R. prolixus* gut microbiota in uninfected stomachs **(A–C)**, intestines **(D)**, as well as in stomachs infected with *T. cruzi* DM28c clone **(E–G)** or Y strain **(H,I)**. The samples were collected at 1 **(A,E,H)**, 7 **(B,F,I)**, 15 **(C,G)**, and 40 **(D)** days after feeding. The numbers represent amplicons for a constitutive gene *rpoB* (1), and genes for T6SSs proteins (ClpV, Hcp1, and DotU1) of *S. marcescens* (2–4, respectively). The letter M represents the 0.5 μg of Thermo Scientific GeneRuler 100 bp DNA ladder, and the symbol (^∗^) indicates the double DNA ladder amount.

In other to confirm if T6SS genes of *S. marcescens* could be transcripted *in vivo* in the digestive tract of *R. prolixus* infected with *T. cruzi*, the tissues were analyzed by RT-PCR from 1 to 40 DAF. Transcripts encoding for T6SSs proteins were detected in triatomines infected or not with *T. cruzi* DM28c clone or Y strains. The *in vivo* gene expression of T6SS was observed by RT-PCR in *R. prolixus* gut microbiota of uninfected stomachs Figures 9A–C, intestines Figure 9D, as well as in stomachs infected with *T. cruzi* DM28c clone Figures 9E–G and Y strain Figures 9H,I. Apparently, samples from triatomines infected with *T. cruzi* showed an earlier and stronger transcription of T6SSs when compared to those from the uninfected group, especially in the case of DM28c clone (7 and 15 DAF, Figures 9F,G, respectively). On the other hand, mRNA for the T6SSs proteins was still detected in stomachs at 15 DAF and intestines at 40 DAF of uninfected triatomines, as showed in Figures 9C,D, respectively.

**FIGURE 10:**
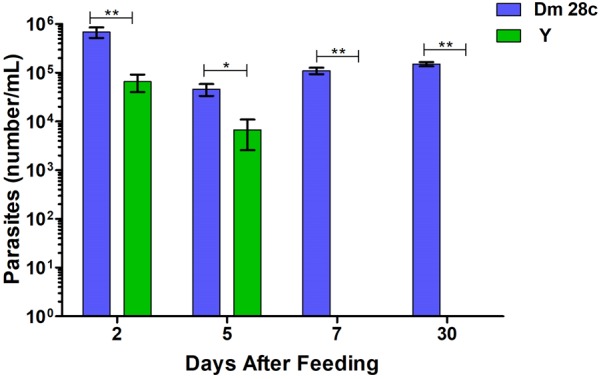
The number of parasites per mL quantified by optical microscope in *R. prolixus* gut in 2, 5, 7, and 30 days after feeding with blood infected with *Trypanosoma cruzi* Dm28c clone (blue) and Y strain (green). Parasites could not be observed in the gut 7 or 30 days after feeding with Y strain. Bars represent the average of parasites in insect digestive tract. Averages were compared using Mann–Whitney test (^∗^*p* < 0.05, ^∗∗^*p* < 0.01).

When counting the number of *T. cruzi* DM28c parasites in the *R. prolixus* TDT, we found it significantly higher compared to that of the Y strain, especially at 7 and 30 DAF (Figure 10, *p* = 0.0079; *p* = 0.0019, respectively).

## Discussion

In this study, we reported on the genomes of two *S. marcescens* strains isolated from the gut microbiota of *R. prolixus* and made available publicly through DDBJ/ENA/GenBank. We analyzed the genomic features of these strains and demonstrated theirs *in vitro* trypanolytic activity against *T. cruzi* as well as the expression of the T6SS *in vivo*.

The study of the intestinal microbiota of insect vectors has focused on the influence of some resident bacteria to interrupt the parasite development within the invertebrate host gut (Azambuja et al., 2004; Cirimotich et al., 2011; Weiss and Aksoy, 2011; Geiger et al., 2013). In Diptera, the bacteria *S. marcescens* isolated from the digestive tract of different species has been proposed as a strategy to prevent *Plasmodium* and *T. brucei* transmission to vertebrates (Rahul et al., 2015; Wang et al., 2017). *S. marcescens* isolated from *R. prolixus* gut possess an *in vitro* hemolytic activity against *T. cruzi (*Azambuja et al., 2004). Here, we found that both *S. marcescens* strains (RPA1 and RPH1) possess genes related to the bacterial hemolytic activity, which could benefit the blood digestion by triatomines. Actually, the trypanolytic activity of *S. marcescens* with regard to *T. cruzi* was previously demonstrated *in vitro* (Azambuja et al., 2004; Castro et al., 2007a,b). Moreover, feeding experiments in which *R. prolixus* was infected with blood containing antibiotics resulted in the reduction of the bacterial population together with the increase of parasites number in the midgut (Castro et al., 2012; Vieira et al., 2016), which indicated the antagonistic effect of *S. marcescens* on *T. cruzi in vivo* (Vieira et al., 2016).

The growth dynamics of bacterial microbiota from *R. prolixus* in liquid medium reaches its maximum around the eighth DAF (Castro et al., 2012). Later, it was shown that the bacterial proliferation activates a basal immune response in *R. prolixus* midgut corresponding to the increase of genes expression of antimicrobial peptides (Vieira et al., 2014), which control the over-proliferation of microorganisms. The trypanolytic activity of *S. marcescens* was previously reported (Azambuja et al., 2004; Genes et al., 2011). Moreover, it was demonstrated that the *S. marcescens* trypanolytic activity is dependent on its adhesion to *T. cruzi* through mannose sensitive fimbriae (Castro et al., 2007a,b; Moraes et al., 2009). Here, we characterized the trypanolytic activity of *S. marcescens in vitro* for the two strains RPA1 and RPH1 with regard to the Dm 28c and Y strains of *T. cruzi*. The prodigiosin (red pigment) production *in vitro* does not seems to be a key determinant of *S. marcescens* antagonism against *T. cruzi* since the lysis induced by RPA1 (non-pigmented strain) was larger than the one induced by RPH1 (pigmented strain).

Concerning adhesive molecules, we found several genes associated to fimbriae, pili, and hemagglutinins in the genomes of both *S. marcescens* strains, which are supposed to be related to their trypanolytic activity. However, only *S. marcescens* RPA1 possessed fimbriae type I (*fimB*) and type IV (*fimA*), which are known to be associated to adherence, invasion, and virulence (Kennan et al., 2001; Pusz et al., 2014). The presence of these genes in RPA1 strain could be related to its stronger trypanolytic activity when compared to RPH1 strain. Moreover, it was found that both *S. marcescens* strains have a PTS-mannose specific related to the uptake of this carbohydrate from the extracellular environment (Postma et al., 1993; Uchiyama et al., 2003). As described in previous publications, the trypanosomatids possess several mannose residues on their membrane surfaces (Bonay et al., 2001; Barboza et al., 2005; Moraes et al., 2008, 2009). In addition, the reported mannose sensitive fimbriae of *S. marcescens* mediate adhesion to eukaryotic cells (Reid and Sobel, 1987). Having taken these pieces of evidence together, we propose that the *S. marcescens* fimbriae play a role in the protozoan recognition by *S. marcescens*, which is supposed to activate a mechanism of incompatibility through the induction of T6SS or other secretion systems. However, further studies are necessary in order to evaluate this cause–effect association.

Type VI secretion system represents an efficient means by which bacteria interact with host organisms or attack competitors. The T6SS of *S. marcescens* Db10, a white entomopathogenic strain, which if directly introduced into the body cavity of *Drosophila* is insensitive to the host’s systemic immune response and kills flies in a day, is active and expressed constitutively under normal growth conditions (Nehme et al., 2007). The T6SS of Db10 strain can assemble itself and be fired without needing to be triggered by cell contact, which broadens its range of cell targets and suggests that the activation of the T6SS is tailored to survival in specific niches (Gerc et al., 2015). Moreover, a multi-stage and dynamic assembly process was observed in Db10 with different subassemblies occurring simultaneously (Gerc et al., 2015). Although some T6SS transcripts have been detected *in vivo*, neither morbidity nor mortality of *R. prolixus* was observed in this study (data not shown). Further studies are, therefore, necessary to clarify if correct assemblies or activations are occurring in *R. prolixus* gut and evaluate whether T6SS is, indeed, involved in the trypanolytic activity observed *in vivo*.

In addition, there have been several recent reports stating that some T6SSs can target bacteria rather than eukaryotic cells (Murdoch et al., 2011). The T6SS has been shown to be involved in the bacteria–bacteria competition of *S. marcescens* with other opportunistic bacterial pathogens, but the role of T6SS of *S. marcescens* in the *R. prolixus* gut is still not completely clear. Although T6SS has been implicated in the virulence of some mammalian pathogens, it is not required for the virulence of *S. marcescens* Db10 strain in three non-mammalian virulence models (Murdoch et al., 2011). It does, however, exhibit dramatic killing activity against several other bacterial species and is required for *S. marcescens* to persist in mixed culture with the opportunist pathogen *Enterobacter cloacae*, which is occasionally found in triatomine gut (Murdoch et al., 2011; Gumiel et al., 2015).

Although the trypanolytic activity of *S. marcescens* was reported in *in vitro* conditions, it was found in this study and others (Azambuja et al., 2004; Genes et al., 2011) that some *T. cruzi* strains successfully colonized triatomine gut even in the presence of *S. marcescens*. Observation from the wild showed that *Serratia, Dietzia, Gordonia, Mycobacterium, Corynebacterium*, and *Rhodococcus* do not ensure protection to prevent triatomines from infection by *T. cruzi* alone or in combination (Gumiel et al., 2015). In fact, bacterial cytotoxic gene expression could vary under distinct physiological conditions, such as digestive tract compartment colonization.

Differences between the two populations of *S. marcescens* isolated from the digestive tract of *Rhodnius* spp. were previously observed using the analysis of 16S rDNA (da Mota et al., 2012) and confirmed here with the genome sequences of two bacterial strains, RPA1 and RPH1. The RPA1 genome shows a high level of similarity with SOLR4 and RSC-14 (two strains associated to plants) as well as ADJS-2D_White (a strain isolated from a phytophagous insect), whereas RPH1 has identity with the aquatic strain 19F (isolated from the skin of *A. zeteki*), WW4 (paper mills use water in their manufacturing process), and EGD-HP20 (from wastewater). The evolutionary divergences between RPA1 and RPH1 isolates were highlighted by comparative genomics and trypanolytic activity as well.

*Serratia marcescens* SOLR4 and RSC-14 are bacteria associated with plants; the first one isolated from an unclassified Solanaceae from Brazil (SAMN08287009) and the second from *Solanum nigrum* (Khan et al., 2015). Besides the hematophagic and coprophagic habits of triatomines (Wigglesworth, 1974; Sandoval et al., 2000), Díaz-Albiter et al. (2016) have also identified the alternative phytophagic behavior of *R. prolixus*, which may feed on *Solanum lycopersicum* fruit and sucrose solution in case of starvation. This finding could explain the close phylogenetic relationship between the strains RPA1 and plant-associated *S. marcescens* strains. Phytophagy by triatomines might enable them to avoid death from starvation in dry environments but might also be the source of endophytic bacteria in their guts.

Similarly, to the digestive enzymes of hematophagous insect, plants include the production of reactive oxygen species (ROS) and reactive nitrogen species, such as nitric oxide (NO) in their defense arsenal (Nascimento et al., 2018). Thus, to be able to colonize internal plant tissues, bacterial endophytes need to be able to cope with these stressful conditions (Nascimento et al., 2018). *S. marcescens* genomes from *R. prolixus* gut encode for various enzymes related to ROS detoxification, including superoxide dismutases, catalases, alkyl peroxidase (only RPA1), peroxidases, peroxiredoxins, organic hydroperoxide, peroxide stress protein YaaA, resistance gene ohrB, and biofilm peroxide resistance protein (BsmA). In addition, many glutathione S-transferase genes, the glutathione ABC transporters, glutathione peroxidases, glutathione synthetases, glutathione reductases, and glutaredoxin genes are found in the RPA1 and RPH1 genomes. A NO dioxygenase gene is present only in RPA1 genome and may account for the strain ability to cope with nitrosative stress. On the other hand, alkylhydroperoxidase and lipid hydroperoxide peroxidase were found only in RPH1.

Gut colonization by symbiotic bacteria requires multiple genomic adaptations since uncontrolled growth could lead to tissue damage and host death. Although the function of pRPA1 and pRPH1 plasmids remains unknown, a previous study showed that the *S. marcescens* colonies maintaining plasmids grow at a slower rate than the plasmid-free once (Platt and Chesham, 1990). Both *S. marcescens* pRPA1 and pRPH1 plasmids share many genes with a high level of identity, including some genes also found in other *S. marcescens* strains isolated from animals (mainly insects), but not observed in plant-associated strains (SOLR4 and RSC-14), suggesting a recent host adaptation niche, despite the evolutionary divergence between RPA1 and RPH1 chromosomes.

The investigation of *Serratia* genomics performed here revealed its adaptation to the life in community with multi-species interactions. The genes involved in motility, chemotaxis, and attachment that have been suggested to play an important role in endophytic bacterial colonization could act in a similar way in the settlement of *Serratia* in triatomine guts. Flagella, fimbria, LPS, and quorum-sensing genes are also abundant in *Serratia* genomes, which is consistent with its ability to sense and colonize several niches, including plants, insects, and nematode (Nascimento et al., 2018).

As previously reported, secretion systems and toxins (Sarris et al., 2014) were found to be encoded by GIs in RPA1 and RPH1 strains. The distinct composition in secretion systems between *S. marcescens* strains may explain their differences in trypanolytic activities. T2SS was observed only in RPA1, which could explain its higher trypanolytic activity compared to that of RPH1. This secretion system was also detected in *S. marcescens* strains Ano1 and Ano2, from *A. stephensi* (Chen et al., 2017), although the mechanism that could interfere with the *Plasmodium* development in the mosquito midgut has not been described yet.

The expression of the T6SS transcripts, found in both RPA1 and RPH1 genome, was also detected in the *R. prolixus* digestive tract. However, the T6SS transcription appeared earlier and stronger in *R. prolixus* infected with *T. cruzi* Dm 28c. The expression of the T6SS was also reported in the *S*. *marcescens* strain WW4 (Chen et al., 2017).

The role played by chitinases in the metabolism of *S. marcescens* is still unclear, but they were detected in RPA1 as well as in RPH1. Partially purified chitinases of *S. marcescens* PRNK-1, an isolated from cockroach, strongly inhibited the fungal growth of *Rhizoctonia solani* and *Fusarium oxysporum in vitro* (Moon et al., 2017). Chitinases secreted in the TDT could be especially important to protect its microbiota from infection by entomopathogenic fungi, i.e., *Beauveria bassiana, Gliocladium virens, Metarhizium anisopliae*, and *Isaria fumosorosea* (Vázquez-Martínez et al., 2014; Forlani et al., 2015; Huang et al., 2016). Although *S. marcescens* possess chitinolytic activity over the fungal wall, it is unknown if it affects the perimicrovillar membrane of TDT which also contains chitin (Alvarenga et al., 2016). The FS14 strain significantly suppressed the growth of phytopathogenic fungi through non-contact inhibition, which might be attributed to the extracellular secretion of chitinases.

Colicins and pyocins are bacteriocins with antibiotic activity secreted by some members of the Enterobacteriaceae that enable them to kill closely related bacterial cells, thereby reducing competition for essential nutrients. Although the antagonistic potential against bacteria has not been evaluated *in vitro* in this study, a whole colicin/pyocin bacteriocin operon including a colicin immunity protein was found in RPH1 (but not in RPA1) next to a PhoP/PhoQ system genes present in all strains (A, B, and C). PhoP/PhoQ is a two-component regulatory system that has been reported to be critical in the virulence control of *S. marcescens* (Barchiesi et al., 2012).

Microbial urease presents the enzymatic property of yielding ammonia, but also carbamate, which is very toxic to the host cells. Urease has been considered as a virulent factor not only for host cells but also for fungi and yeast (Giannouli et al., 2014). In presence of the *S. marcescens* urease, the urea from ingested mammalian blood might generate ammonia, which might contribute to nitrogen bioavailability and increase pH in midguts of hematophagous insects. This mechanism of pH control was proposed in *A. stephensi* (Chen et al., 2017) and might be expanded to other insect vectors such as *R. prolixus*. In the present study, a complete cassette encoding genes for urease was detected in *S. marcescens* RPA1 and RPH1 genomes.

The acquisition of plasmids is a major factor in the ability of bacteria to exploit new environments and hosts, including human and Hemipteran insects (Ricci and Hernandez, 2000; Douidah et al., 2014; Perilla-Henao and Casteel, 2016). Some *Serratia* strains are considered opportunist pathogens for human and some virulence genes are frequently described in their genomes, mainly in plasmids (Hurst et al., 2000; Iguchi et al., 2014; Gruber et al., 2015). pKPC-56ce, a plasmid similar to those of pRPA1 and pRPH1, was found in the SSNIH1 strain of *Serratia* sp. isolated in 2015 from a hospital tubing in the United States (Weingarten et al., 2018). Fortunately, pRPA1 and pRPH1 plasmids do not include the integron found in pKPC-56ce and its resistance genes for quinolones (QnrB19, C3F38_25970), betalactams extended-spectrum (SHV-12, C3F38_25945), and carbapenems (KPC-2, C3F38_25920). On the other hand, pRPA1 plasmid encodes the aadA1 gene (B7L32_25510) for aminoglycoside resistance also found in pKPC-56ce, but not in pRPH1.

The coprophagic behavior of triatomines might explain the variability of *S. marcescens* populations in the digestive tract of specimens from the wild. Gumiel et al. (2015), analyzing the microbiota diversity in the digestive tract of *T. brasiliensis* and *T. pseudomaculata*, also observed the presence of *S. marcescens* in all specimens captured in the endemic northeastern region of Brazil. In addition, Gumiel et al. (2015) also reported the sporadic presence of *Rhodococcus*, which belong to the *Corynebacterineae* suborder as well as other bacterium genera. Moreover, Carels et al. (2017) noted that all these bacterial species share one common feature: bacteria with GC-rich genomes overcome those with GC-poor genomes. These authors attributed the comparative success of GC-rich bacteria in the TDT niche to their increased ability to process complex substrates.

In this study, Actinobacteria such as the symbiont *R. rhodnii* were not detected, probably due to the short time of incubation on BHI agar. In fact, the TDT microbiota diversity is probably underrepresented in this study due to limitations of *in vitro* culture and the fastest growth of *S. marcescens*. Despite its apparent predominance, the limitations on molecular fingerprint methods could sub represent the genetic diversity of *Serratia* populations, since only one genetic marker (one fingerprint primer) was applied to classify the isolates (da Mota et al., 2004, 2012). Interestingly, the general distribution of *S. marcescens* in triatomines (da Mota et al., 2012; Gumiel et al., 2015) and mosquitoes (Wang et al., 2017) seems to be a favorable factor to propose it as a suitable competitor species in these systems. However, *S. marcescens* does not seems to be antagonistic of *Wolbachia* sp., since this bacterium is intracellular and has already been observed in co-colonization with *Serratia* sp. in a sylvatic adult specimen of *Rhodnius* sp. from Amazon, Brazil (da Mota et al., 2012). At least two types of *Wolbachia* sp. were also detected in specimens of *Rhodnius pallescens* raised in insectarium and captured in the field in Republic of Panama (Espino et al., 2009).

Although this study presents the first pieces of evidence of an antagonistic potential of *S. marcescens* with regard to *T. cruzi* in *R. prolixus* based on the genomic characterization of RPA1 and RPH1 strains, further investigations are needed to improve our understanding of this system in triatomines.

## Author Contributions

FM, DC, CV, MG, and PA conceived and designed the experiments. FM, DC, CV, and MG performed the experiments. FM, DC, CV, JA, NC, and PA analyzed the data. FM, DC, CV, and PA contributed reagents, materials, and analysis tools. FM, DC, CV, JA, NC, and PA wrote the manuscript.

## Conflict of Interest Statement

The authors declare that the research was conducted in the absence of any commercial or financial relationships that could be construed as a potential conflict of interest.

## Supplementary Material

TABLE S1 Average nucleotide identity between Serratia genomes, bacterial secretion systems, KEGG and Ano1 orthologous, gene islands, antibiotic resistance genes and one way ANOVA of trypanolytic activity.
